# Poised for Change: University Students Are Positively Disposed toward Food Waste Diversion and Decrease Individual Food Waste after Programming

**DOI:** 10.3390/foods10030510

**Published:** 2021-03-01

**Authors:** Manar A. Alattar, Jennifer L. Morse

**Affiliations:** 1 Department of Environmental Science and Management, Portland State University, Portland, OR 97201, USA; jlmorse@pdx.edu; 2 Department of Environmental Studies, University of Portland, Portland, OR 97201, USA; 3 Department of Biology Portland, Portland Community College, 12000 SW 49th Ave., Portland, OR 97219, USA

**Keywords:** wasted food, food waste, waste diversion, cafeteria programming, cafeteria intervention, environmental behavior, behavior change, sustainability, food systems, climate change

## Abstract

Eaters (consumers of food) are responsible for 60% of waste along the food cycle in developed countries. Programs that target individual and household food waste behavior change are essential to addressing such waste. School cafeterias worldwide offer an opportune microcosm in which to educate on food and nutrition skills and change related behavior. No Scrap Left Behind, a cafeteria food waste diversion program, was developed, piloted, and assessed based on measures of both direct and indirect food waste behavior, and attitudes, knowledge, and emotions related to food waste. Participants had positive attitudes towards food waste reduction, engaged in food waste diversion actions, had some knowledge of the impacts of wasted food, and considered their actions important to waste reduction generally. Food waste per student was decreased by 28% over the course of the first year of programming (*p* = 0.000967), and by 26% in the following year when measured a week before and a week after programming occurred (*p* = 0.0218). Results indicate that students were poised for food behavior change and that related programming did impact behavior in the short term. Programming may, therefore, help improve student attitudes and skills to develop long-term change as well, although future research should explore this specifically. In comparison with other research on cafeteria programming, results suggest that food waste diversion programming can positively impact students’ dispositions and behaviors, and may be more effective when tailored to the specific population.

## 1. Introduction

Wasted food is a symptom of inefficiencies in the use of human, natural, economic, and political resources globally during food production and consumption [1,2]. Food system inefficiencies must be addressed holistically, and education is one important tool among others for decreasing food waste and improving the footprint and overall justice of the global food system [3,4]. Educational institutions worldwide have recognized their ability to encourage food waste reduction and improve their own measures of institutional sustainability through campus food diversion programming [5,6,7,8]. In university cafeterias in the United States of America (USA), 3.6 million tons of food is wasted annually [9]. Waste audits of university campuses identify food, representing one-fourth or more of all campus solid waste in some cases, as a primary opportunity for solid waste reduction [8,10]. Most often, plate waste includes starch components, fruits and vegetables, and other side dishes [11].

The issue of food waste is not unique to higher education. An estimated 26% of food offered through federally funded national school lunch programs is wasted. In the USA, this results in an estimated loss of $1.2 billion annually [12], double the estimate in 2002, from national school lunch funding [7]. Research on school cafeteria food waste from around the world has shown that students produce between 51.3 to 121.9 g of food waste per meal (usually lunch) (Table 1) [6,7,13,14,15,16,17]. Plate waste is particularly concerning in the case of school children, who have been frequently shown to consume insufficient amounts of calories, fiber, vitamins, and minerals from school lunches [12].

Research on methods for decreasing food waste in schools is emerging [19]. Cafeterias that have implemented tray-less dining decreased their food waste generation by approximately 30% [20]. Item-by-item sale (as opposed to open buffet) decreased waste by 14% [13,21]. Plate size has also been shown to correlate positively with food waste [22]; as a result, many cafeterias have decreased the size of plates offered at buffets. These are all examples of behavioral nudges, in which behavior is influenced through subtle changes to the environment, rather than direct behavior intervention [23,24].

Interventions that target attitude and behavior change directly have also been shown to decrease food waste [18]. Simple, informative campaigns achieved a 15% reduction in food waste [16,18]. A program including interactive food waste messaging, both in and out of the cafeteria, and food waste buffets, to display the accumulation of student food waste, at University of California, Davis (UC Davis, Davis, CA, USA) even achieved a 50% reduction in food waste after seven years of programming [15] (Table 1). As eater (as opposed to “consumer”) education has been identified as a primary solution to global food waste, research developing and assessing educational food waste diversion tools is an essential next step towards decreasing food waste [3,21].

In addition to reducing overall campus waste, efforts in school cafeterias have the potential to influence long-term food waste behavior of students. Firstly, due to the number of meals many students eat in school cafeterias, this environment has lasting effects on their eating and health behaviors [25]. Secondly, secondary and post-secondary education often are times of identity development and formation, which impacts behaviors throughout life [26]. Finally, cafeterias, like laboratories, allow for experimental manipulation that can encourage learning and behavior change in students, such as in the example of behavioral nudges.

In the USA, as of September 2015, goals for 50% food waste reduction by 2030 have been set by U.S. Environmental Protection Agency (EPA) and United States Department of Agriculture (USDA) [27]. Many universities similarly have climate actions plans that address food waste. Our research was conducted at Portland State University, one such university in Portland, OR, USA. Food waste represents 25% of the campus waste stream [10] and the institution is working towards 25% waste generation reduction and 10% landfill-bound waste reduction by 2030 (Portland State University Climate Action Plan). To help contribute to these goals, a broad assessment of student attitudes, emotions, and reported behaviors was completed [28] and a food waste diversion program, No Scrap Left Behind, was developed, piloted, and assessed. Program assessment was based on both direct and indirect measures of behavior and dispositional factors.

In order to assess the program, food waste behaviors were measured directly by weighing food waste and indirectly through surveying. Surveys also measured reported knowledge, attitudes, emotions, and beliefs related to food waste diversion and sustainability, which are important contributors to food related behaviors [29]. The objective of the program was to decrease food waste production per student in the cafeteria and assess changes in students’ knowledge, attitudes, emotions, and behaviors toward food waste.

We hypothesized that, after a year of No Scrap Left Behind programming, food waste production per student per lunch would decrease, and student knowledge, attitudes, emotions, and reported behaviors related to food waste would improve from the beginning of the program to the end.

## 2. Methods

### 2.1. No Scrap Left behind Program

**Figure 1:**
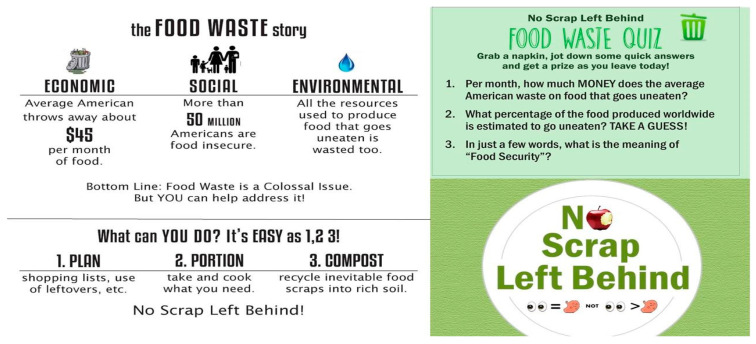
Examples of No Scrap Left Behind signage. Signage focused on economic, social, and environmental impacts of Figure 2. As students left the cafeteria, surveys were collected in exchange for a small prize (choices included reusable spork-knives, pens, mini-first aid kits, and candy). Quizzes, signage, surveys, and the food waste buffet were all opportunities to encourage student engagement in and interaction with program and program volunteers.

The No Scrap Left Behind program was developed to increase student awareness of the social, economic, and environmental impacts of wasted food (see Supplemental for program file). The program tagline was “Plan, Portion, Compost” to clearly promote a sequence of actionable food-related tasks (Figure 1). The program included informational discussion tabling with trained volunteers and signage throughout the cafeteria (Figure 1; Supplemental). Signage considered the economic, environmental, and social impacts of wasted food, with a slight bias towards economic impacts, which have been shown to be important influencers of behavior [3,18,30]. Cafeteria napkin holders also included brief fun and actionable messaging and a Food Waste Quiz (four versions throughout cafeteria) that students could answer on a napkin and bring back to the program table for a prize (Figure 1, right panel).

Both actual and reported (survey-based) food waste data was collected at the start of (baseline) and throughout programming. Food waste totals were displayed on a board in the cafeteria at the end of each program day. Research on proenvironmental behaviors suggests that increased visibility of the issue or related action is more likely to lead to proenvironmental action [31].

### 2.2. Study Location and Sample Population

Survey data and food waste measurements were collected during food waste diversion programming at a university cafeteria in the USA. Programming occurred during lunch period for one week each term for three semesters (fall, winter, and spring), then again, a year later. Food waste weights were collected each term during programming, and in the initial and final terms in the kitchen (blind to students) as a control. Survey data was also collected in the initial and final terms.

**Figure 2:**
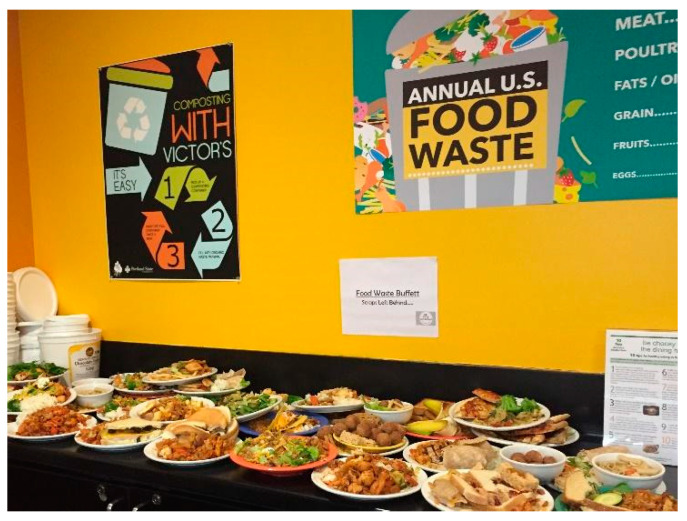
A food waste buffet was curated by volunteers in the tray return area of the cafeteria as students completed their lunch. This allowed for the clear visualization of the accumulation of wasted food over the lunch period. It also allowed for interaction and discussion between students and program volunteers.

The cafeteria hosts about 300–400 people at lunch each day [32]. A total of 174 surveys were collected through convenience sampling in the Fall (initial) and Spring (final) terms. Students were given surveys while waiting in line to pay for lunch or while eating, and they returned their completed surveys after their meal. Food waste was curated for students to observe and weighed during the programming week (Figure 2).

### 2.3. Survey

Respondents were asked to report on knowledge, attitudes, emotions, beliefs, and behaviors related to food waste in 30 Likert-type questions and three written-answer questions, similar to the survey instrument used in Alattar et al. [28]. All Likert-type questions were given a five-point response scale that ranged from “Strongly agree” to “Strongly disagree”, with “Neutral” as the middle anchor point. A 5-point scale allows for sufficient variation within the scale without risking participant reluctance to choose extreme answers on a wider scale [33]. Questions were asked in both pro-food waste diversion form (e.g., “I eat leftovers”) and antifood waste diversion (“Food waste doesn’t bother me”) to diversify and capture a broader range of responses. Questions written in antifood waste diversion form were reverse-coded for analysis, which is common in such research [34,35]. Cognitive interviews were conducted with a number of potential respondents and survey experts to establish the content validity of the instrument prior to data collection.

Food waste knowledge was measured with questions that have been used in other food waste studies [36,37] and questions on specific campus-related food waste diversion knowledge [16,38]. “I understand food freshness labels (sell by, best by, use by, expiration date, etc.)”, and “I know about the campus composting program” are examples of general and specific, respectively, food waste knowledge items. Knowledge was also probed by asking respondents to estimate the percent of food waste at various consumer levels: average American household, the campus community, and the USA. as a nation, and along the food cycle from production to consumption. Direct questions about the amount of food participants wasted (as a percentage of total food).

Intent and interest in food waste reduction was measured with questions including “I put effort into reducing food waste” and “I am interested in taking action to prevent food waste”, as done in or suggested by other work [19,39,40]. Food management skills have been cited as important to food waste generation [40,41,42] and were measured using a series of questions similar to those in a recent national survey [40] like “I eat leftovers”, “I check the refrigerator before shopping”, and “I compost my food scraps.”

Attitudes towards food waste were measured with both cognitive and affective statements, including “Food waste does not bother me”, “My individual actions towards food waste do not make a difference”, “Composting stinks and is gross”, and “When I compost I feel like I’m contributing to the greater good” [40,43,44]. Perceived cost of food waste was measured with two items, “I don’t think the food I throw away costs much money” and “When I go to a buffet restaurant, I take more than I can eat to get my money’s worth.”

Broader sustainability beliefs were probed indirectly with the following questions: “I believe that many materials can be reused or recycled into something new”, “I believe proper waste disposal makes a positive environmental impact”, “I would like to see more programs that help reduce food waste”, and “I would enroll in a course with a sustainability theme”. Questions specific to the university cafeteria were asked as well; one asked about satisfaction with the food served by the dining hall, and the other three were related to knowledge and usage of cafeteria composting and reuse options. Basic, university-related demographics were also collected, including age, gender, academic level, and whether students lived on-campus.

### 2.4. Food Waste Buffets and Compost Audits (Direct Measurement of Behavior)

This study combines both direct (food waste buffet and compost audits) and indirect (surveys) measures of behavior in response to programming. Other studies have tended to focus on either directly quantifying food waste [6,17] or surveying [40], although some have done both [16,45]. The combination of direct behavior measurements with survey data provides evidence of whether behavior is actually being affected, rather than relying on self-report data [41,46]. In our study, survey results and measures of food waste were not linked directly to individual students. Instead, changes in overall survey results and overall food waste quantities were tracked and reported on.

Surveys included indirect, self-report measures of student behavior, whereas direct student food waste behavior was measured in two ways:

(1) Food waste buffet weights—During the No Scrap Left Behind programming, food scraps were collected from all students during two hours of lunch period. The cafeteria does not have any disposal containers available to the students; rather it has a single revolving tray return at the exit. Food was collected at the tray return, curated by volunteers into a “Food Waste Buffet” (Figure 2), and weighed at the end of lunch. Food scraps were collected and weighed separately from napkins, fruit rinds, and other inedible compostables. Liquid volumes were not collected.
(2) Kitchen audits—The possibility of social desirability bias in the measured food waste was significant [47]. In other words, students could be wasting less food because of the presence of the No Scrap Left Behind volunteers and social pressure from the programming. Therefore, food waste weights were measured in the kitchen (where students could not see that it was being done) in a single week following programming. These weights included inedible compostables, which were later subtracted out based on the average percentage of inedible compostables from the program weeks. In the Winter of 2017, these weights were measured in both the week before and the week after programming for comparison.

All food waste weights, direct or indirect, were determined using the average weight of food waste production over a week per the average number of customers served on those days (data provided by the cafeteria).

In order to control for student acclimation to the cafeteria system and its food options, which may inherently decrease amounts of food waste over time, we compared changes in food waste both over an academic year and within an academic term the following year (only food weights; no surveys assessed for follow up year). This allowed us to confirm that changes in food waste were seen both within the long term (over an academic year of programming) and the short term (directly after programming). Parallel changes in both time frames would point to the programming as the main contributor to such change, whereas changes over the year and not directly after programming within a term would indicate that other factors may have contributed to the changes in food waste behavior.

### 2.5. Data Analysis

The program was first run in the 2015/2016 academic year; it has continued to run in the cafeteria since. Survey responses from the first year of programming were used to assess the initial impact of the program on behaviors and attitudes related to wasted food; initial data from Fall 2015 was compared to data from the end of the first year of programming (Spring 2016). In order to confirm that the changes in food waste weights seen in the initial year were due to the program, rather than other factors, food waste weight data was additionally collected to compare student food waste production and attitudes within a single term in the second year of programming (comparing data from the beginning and end of Winter 2017).

Survey data questions included both response and Likert scale items. Although Likert items may not meet *t*-test assumptions of normality and are not continuous, research has shown that *t*-tests are acceptable and appropriate for comparing Likert items [48]. For direct measures of behavior (food waste buffet and kitchen weights), average food waste per student was calculated based on customer transaction numbers for the programming period. A significance threshold of 0.10 was most appropriate for this research due to the complexity of interactions between variables related to human behavior and to reduce the chance of false negatives within such a small sample size [49].

## 3. Results

### 3.1. Sample Characteristics and Demographics

From the 300–400 students that eat daily at the cafeteria, we collected a total of 174 surveys through convenience sampling at the beginning (Fall 2015; *n* = 88) and end (Spring 2016; *n* = 86). The average age of respondents was 20 years old, with a range of 18–38 years. Of participants, 47% were female and 49% male. A majority (91%) lived in residence halls on campus. On average, participants ate at the cafeteria 10 times a week. Participants lived in dorms/houses with an average of two residents per household. No survey data was collected in Winter 2017, only food waste weights data.

### 3.2. Food Waste Buffet and Kitchen Audit Data (Direct Behavior Measurements)

**Figure 3:**
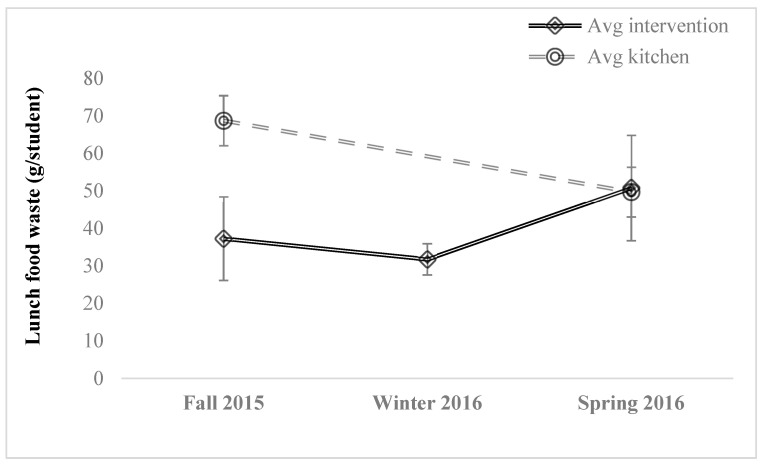
Trends in student food waste (g/lunch) as measured at the food waste buffet (in view of students) and compost kitchen weights (out of sight of students) over the year of programming (Fall 2015 to Spring 2016). Data was collected by recovering all wasted food during lunch for a week and taking the average of that waste based on customer number data (provided by the cafeteria). Standard deviation indicated with error bars.

Students produced an average of 68.78 g/student of wasted food at the program onset, as measured in the kitchen (Table 2). This amount of food waste was similar to many previous studies (Table 1). When the Fall 2015 programming volunteers were present, food waste was drastically lower, 37.29 g/student. We attributed this to social desirability bias [50] and relied on kitchen weights for program assessment. As the programming year concluded though, kitchen weights were similar to weights measured during programming (when volunteers were visible), and were 49.71 g/student (kitchen) and 50.81 g/student (during programing) respectively (Figure 3).

As predicted, student food waste based on kitchen audits decreased significantly by 28% within one academic year (Fall 2015 to Spring 2016; one-tailed *t*-test, *p* = 0.000967). However, it is evident that student food waste may decrease over an academic year as students become more accustomed to the food and cafeteria settings. To control for this, food waste data was also collected in the kitchen (out of sight of students) in the following year in the Winter 2017 before and after No Scrap Left Behind programming that term. Still, food waste decreased from an average of 64.3 to 87.0 g/student, a 26% decrease, within one term of programming (Winter 2017; one-tailed *t*-test, *p* = 0.0218) (Table 2; Figure 3). Social desirability bias seemed still pertinent as the food waste weights during programming were much lower, 41.0 g/student.

### 3.3. Survey Data

Student responses were compared from the beginning of the programming year (Fall 2015) to the end of the year (Spring 2016). Overall, students began the programming year with positive knowledge, attitudes, emotions, and beliefs related to food waste diversion, similar to the preliminary research performed more broadly across campus [28]. Participants had some awareness of the issue of wasted food. For example, on average, students thought the USA. wasted 51% of food produced and that the average American wasted 36% of the food they purchased. Students had positive attitudes towards food waste diversion. For example, 88% agreed that materials should be reused and recycled, and 90% agreed that proper disposal was essential to reducing environmental impacts. Participants reported putting effort into food waste reduction (62% agreed or strongly agreed), thinking about the food waste they generated (65% agreed or strongly agreed), and believing that their actions made a difference (69% agreed or strongly agreed). Participants reported engaging in various food waste reduction behaviors. For example, 76% ate leftovers, 80% checked their refrigerator before shopping, 63% made a shopping list before shopping, and 32% composted their food scraps.

Yet, when fall survey data was compared to that from the end of the program, there were few questions in which significant differences were detected. Specifically, students were 11% more likely to agree that “I think about the food waste I generate” at the onset (65%) compared to the end (76%) of the programming year (one-tailed *t*-test, *p* = 0.0382). Students were also 10% more likely to agree that “I put effort into reducing food waste” at the beginning (62%) compared to the end (72%) of the year (one-tailed *t*-test, *p*-value = 0.0487). No other significant differences were detected in survey responses.

## 4. Discussion

Wasted food, an indicator of systemic food system issues, is pervasive and essential to address. Educational programming, among many other things, is an essential aspect of improving food-related behaviors. Programming implemented at university campuses has been shown to impact both student dispositions towards food systems, and actual wasted food [16,18,21]. In this study, cafeteria programming that included messaging, discussion tabling, interactive activities, and stark visual examples of wasted food (food waste buffet, Figure 2) was correlated with a decrease in student food waste by between 26% and 28%. We found that food waste data collected in the kitchen, without student knowledge, was more reliable than data collected during programming with student knowledge. We suggest that this is due to social desirability bias. Studies that measured waste only during programming and saw only moderate changes in food waste after programming may have detected a greater impact if control data was collected more discretely (Table 1).

In our population, attitudes and dispositional factors related to reducing food waste were already high [28] and changed relatively little after programming. Still, students were significantly more likely to think about food waste reduction (10%, *p* = 0.0382) and to put effort into food waste reduction (11%, *p* = 0.0487), by the end of the programming year. A similar outcome was found in a study by Whitehair et al. [16] in which informative cafeteria messaging decreased food waste but had little impact on already positive sustainability beliefs. In contrast, some studies that found initially low support for sustainability or food waste understanding/attitudes, also found modest improvements in food reduction [21]. Other studies showed improved attitudes and decreased food waste [18]. These various findings indicate the impact of programming generally, but also the importance of tailoring interventions to specific student populations. Since students in our study already had some understanding of the impacts of wasted food and positive attitudes and emotions towards food waste reduction, they may have been prepared to make behavioral changes with the correct programming. Research also indicates that behavioral change in adults (particularly in the short term) can often be easier than changing attitudes [39]. At least in the short term, social pressure from programming also likely affected food waste behaviors [31].

We were aware that the decrease in food waste could also be related to students’ increasing familiarity with the food and cafeteria over the year of programming. Since PSU only has one residence hall cafeteria, we could not run a parallel control for this. Instead we confirmed that food waste behavior was also influenced by programming within a single academic term in the year following the initial data collection. It can be assumed that if food waste decreases in the week directly after programming compared to the week directly before, then the program is more likely a key contributing factor rather than gradual acclimation to the cafeteria system. Student familiarity with the cafeteria can be assumed to be relatively similar within those couple of weeks. Additionally, this second round of data was collected in the middle of the academic year (winter term) to control for the many changes that students experience initiating (fall) and concluding (spring) an academic year. Therefore, the significant decrease (26%, *p* = 0.0218) in food waste within a single term (Table 2) suggests that the program was effective regardless of student acclimation to the cafeteria.

Food waste as measured during programming, in front of the students, was initially lower than weights measured behind the scenes, in the kitchen. Results from the two measurement approaches became similar by the end of the year. These results suggest that social desirability bias likely impacted student food waste behavior when they were first introduced to the No Scrap Left Behind program and volunteers [50]. Since kitchen weights and program weights were essentially the same by the end of the year, it can be assumed that the effects of social desirability bias tapered off as students became more familiar with the program and its volunteers over the year. Anecdotally, volunteers also reported that students were being more cautious of their waste during programming days, especially at the beginning of the year. It was noted that some students brought food to the tray return that seemed to be intended for waste and finished it quickly before turning in their plate to volunteers.

High turnover in cafeteria staff and management personnel was a notable challenge, especially in food waste measurements in the kitchen (conducted by cafeteria staff). This is a ubiquitous issue for most food programming, as hospitality industries, including hotel and restaurant employees, have some of the highest turnover rates of all industry categories (highest of all measured industries in 2016; 28.6%) [51]. In order to compensate for such turnover, aspects of the program should be incorporated into the food service company’s sustainability practices, and more frequent trainings should occur with cafeteria staff and management personnel about the programming. In fact, research shows that although the contracting body (the university in this case) can include sustainability practices within the contract with the food service agency, such practices are more likely to succeed when they are already built into the policies of the food service agency itself [11,52]. In fact, in this case, food reduction initiatives were an important component of the contract negotiations that led to a change in dining service management at this university.

Going forward, the No Scrap Left Behind program design is continuously being improved, as it is now standard programming in the cafeteria. Enhancements include more social media connections, a food waste pledge to encourage student commitment to food waste reduction, more interactive programming including film screenings, panels and other out-of-cafeteria events, and more student feedback and discussions related to food waste.

## 5. Conclusions

Student food waste generation decreased by over one-fourth both within one term and over one year of the No Scrap Left Behind programming. Students’ knowledge, attitudes, emotions, and reported behaviors related to food waste reduction were relatively positive. Few significant changes were seen in survey responses over the programming period, although students reported being more likely to think about and put effort into food waste reduction. Other cafeteria food waste reduction programming studies report changes to dispositional factors with little change in behavior, and some have reported improvements in both dispositional and behavioral factors [15,18,21]. It can be concluded that food waste reduction programs have an impact on students, but at varying levels depending on the program and student population. Such findings are encouraging and have resulted in the establishment of food diversion programs in many university cafeterias. No Scrap Left Behind programming, specifically, has been run every year since the initial pilot reported on here. Research on the overall impact of programming over many years would be valuable in understanding the long-term impacts of such programming on sustainability and food related behaviors. The results of this study and others suggest the great potential of university food waste diversion programming for impacting student (and hence more generally, citizen) food waste behaviors.

## Figures and Tables

**Table 1:** Food waste per student as reported from various cafeteria food waste studies. Studies listed from most recent to oldest. The table presents the average waste per student per meal as reported in various school-based food waste studies. When an intervention was conducted, it is summarized along with the initial weights, final weights, percent change, and meal measured.

Study Setting	Intervention Type	Average Waste (g/Student)	Waste after Intervention (g/Student)	Percent Change	Time of Waste Collection
University of Lisbon, Portugal [18]	Informational posters and student separation of organic/inorganic waste in cafeteria	76.5 (meal and soup)	64.7 (meal and soup)	−15%	Lunch
Florida (3 grade schools—public and private) [17]	(No intervention)	52.2 (13% of total waste)		N/A	Waste per school day
UC Davis [15]	Extensive annual programming, discussion tabling, signage, and food waste buffet table	102.06 (year 2009)	51.31 (year 2016)	−50%	Lunch
Kansas State University [16]	Informational cafeteria signage	57	Various	−15%	Lunch (per tray)
Western Michigan University [13]	Compared item-by-item sale and tray-less dining as alternatives to all-you-can-eat	121.90	104.90 (item-by-item sale)$$82.21 (tray-less)	−14% (item-by-item)$$−33% (tray-less)	All day (breakfast, lunch and dinner)
University of Jordan [6]	(No intervention)	70		N/A	Lunch
Virginia Polytechnic Institute and State University [14]	Tray-less dining	117.03 (with tray)	88.90 (tray-less)	−24%	Food collected the whole week (average of all meals)
Various Boston Middle Schools [12]	(No intervention)	(26.1% of total food)		N/A	Lunch
Nationally representative school data (1991–1992) [7]	(No intervention)	(various studies report 10% to 37%, but 12% most reliable)		N/A	Breakfast and lunch

**Table 2:** Comparison of average food waste per student over an academic year of programming and within a single term. Data collection and comparison occurred within the week of programming and in the kitchen (out of sight of the students) the week before and/or after programming in order to control for social desirability bias.

		Initial (g/Student)	Final (g/Student)	% Change	*p*-Value
Year (2015–2016)	During programming	37.29 ± 11.19	50.81 ± 14.09	36%	6.67 × 10^−2^
	Kitchen	68.78 ± 6.65	49.72 ± 6.68	−28%	9.67 × 10^−4^
Term (Winter 2017)	During programming	40.97 ± 7.09			
	Kitchen	87.03 ± 14.39	64.27 ± 13.31	−26%	2.18 × 10^−2^

## Data Availability

The data presented in this study are openly available at https://sites.google.com/a/pdx.edu/morse-lab/publications (accessed on 2 June 2017).

## Supplementary Materials

The following are available online at https://www.mdpi.com/2304-8158/10/3/510/s1.
