# Supplementary material for: Poised for Change: University Students Are Positively Disposed toward Food Waste Diversion and Decrease Individual Food Waste after Programming
Source: Foods. 2021 Mar 1;10(3):510. doi: 10.3390/foods10030510 (PMC7998915; doi:10.3390/foods10030510)
Supplement: Supplementary file 1 [file foods-10-00510-s001.pdf]

Saving the Food: Saving the Planet

Waste diversion programs appendix

Summary: These programs were developed through work by Manar Alattar, PhD and partners at Portland State University to promote food diversion through educational programming on campus.

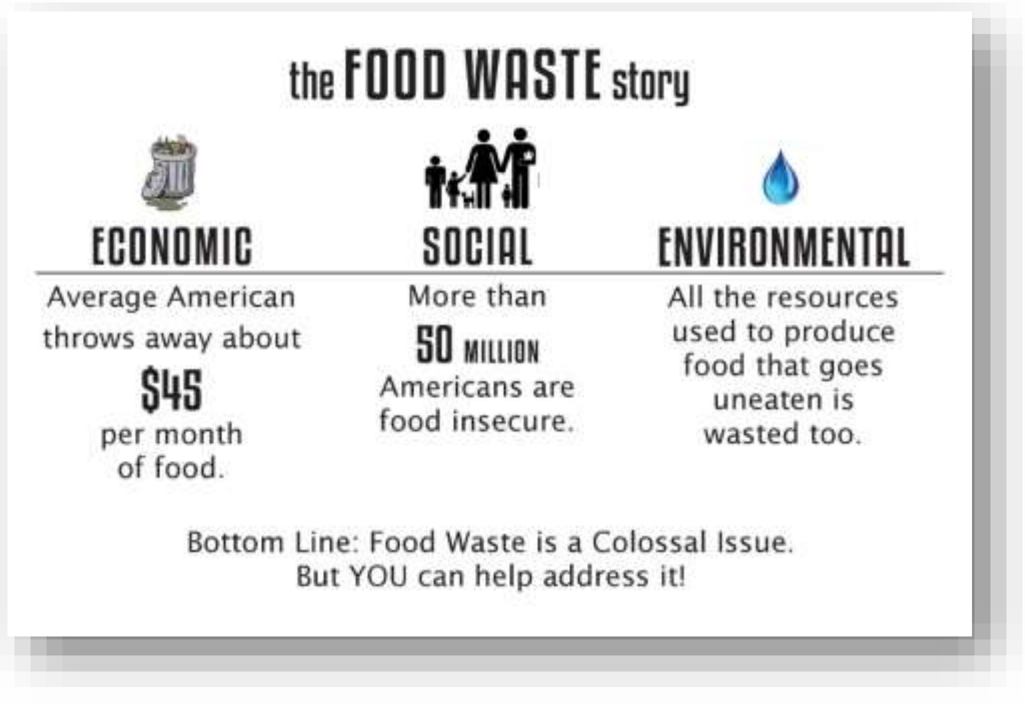

Diagram credit: Holly Carman-Fujioka

Table of Contents

Appendix A. *No Scrap Left Behind* toolkit 4

Appendix A.1. *No Scrap Left Behind* Toolkit Guide 4

Appendix A.2. Program signage 8

Appendix A.3. Student handouts 23

Appendix A.4. Surveys 28

Appendix B. Food waste photo gallery program 33

Appendix C. Food waste mindful cook-off activity 37

Appendix A: *No Scrap Left Behind* toolkit

Appendix A.1. *No Scrap Left Behind* Toolkit Guide

Introduction:
No Scrap Left Behind is a food waste diversion program designed and piloted at Portland State University (Portland, OR) based on programs at other universities including the University of California, Davis *Love Food, Don't Waste* program. *No Scrap Left Behind* is designed to engage students in active learning around food waste and food waste diversion skills. The program seeks to engage students in food waste diversion in relation to the economic, social, environmental, and health impacts that it has. The program also seeks to help students develop some basic skill around food portioning and food waste diversion. Students participating in the program are surveyed (convenience/snowball sampling) about knowledge and behaviors around food waste, both to open the discussion and assess the program. The program success is further measured by measuring the amount of student generated food scraps composted during lunch the week prior to and after the week of the intervention each term. This allows us to determine the effect of the program/volunteer presence in the cafeteria and whether students change their behavior in response to us or intrinsically, long-term.

Objectives:
1. To engage students in **food waste/portioning awareness educational programming** as they pass through the cafeteria and dispose of their waste.
2. To **assess student change in knowledge and behavior related to food waste/portioning** by comparing pre-and post-participation survey results and food scrap weights.
3. To help inform catering services about **potential opportunities for economic savings by encouraging student food waste reduction**.

Process:
The *No Scrap Left Behind* cafeteria intervention was run once a week each term of the academic school year. The cafeteria (wall posters, service stations, tray return, napkin holders, *etc.*) were re-signed for the intervention to promote and inform around food waste diversion. Volunteers tabled to discuss and interact with students about food waste and portioning. As students came through to the tray return area their food waste was collected and curated into a food waste buffet.

Program materials:
Full program materials can be found HERE. These materials were developed in collaborating offices at Portland State University, including the Campus Sustainability Office (CSO), PSU Dining, and the Center for Student Health and Counseling (SHAC). These contributors reserve the rights to grant access to their

materials selectively to specific institutions. We also request that the program be cited in any formal or informal publications about the program. All marketing material file names in this toolkit are labeled with the office in which they were developed.
Molly Bressers (Program & Outreach Coordinator at CSO), Holly Carman-Fujioka (PSU Dining Marketing Coordinator), and Hannah Heller (SHAC) developed marketing materials. Manar Alattar (CSO) supervised the program and material development overall. Anthony Hair (CSO) along with many dedicated volunteers also helped supported and implemented the program.

Note: The crying food images on the medium sized posters are by the Love Food Hate Waste campaign (LoveFoodHateWaste.com) and are cited directly to them.

For questions, comments or material requests, please contact Manar Alattar at manaraalattar@gmail.com.

Photo Gallery

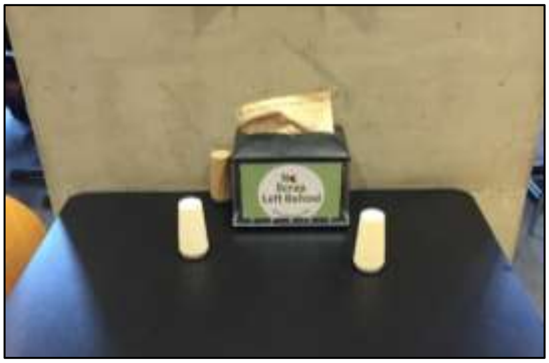

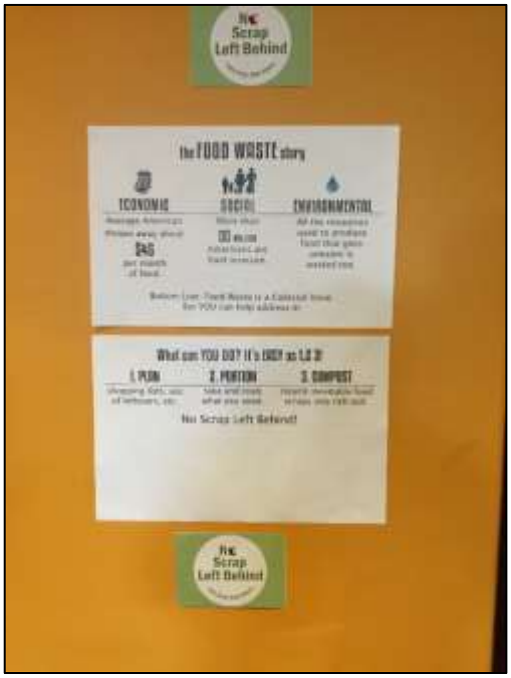

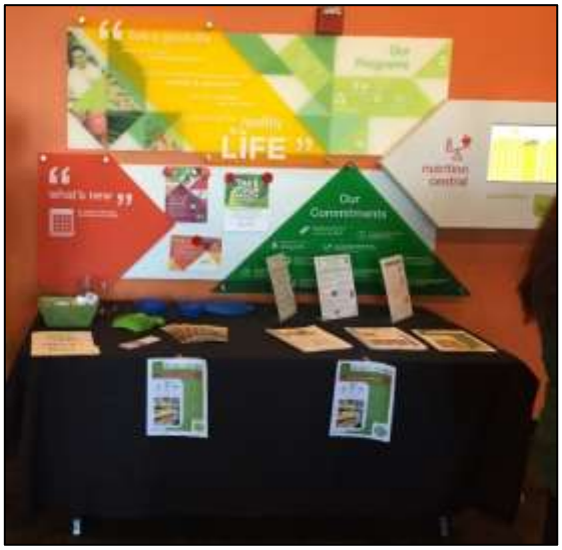

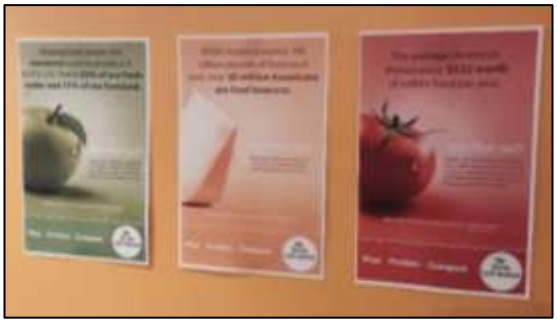

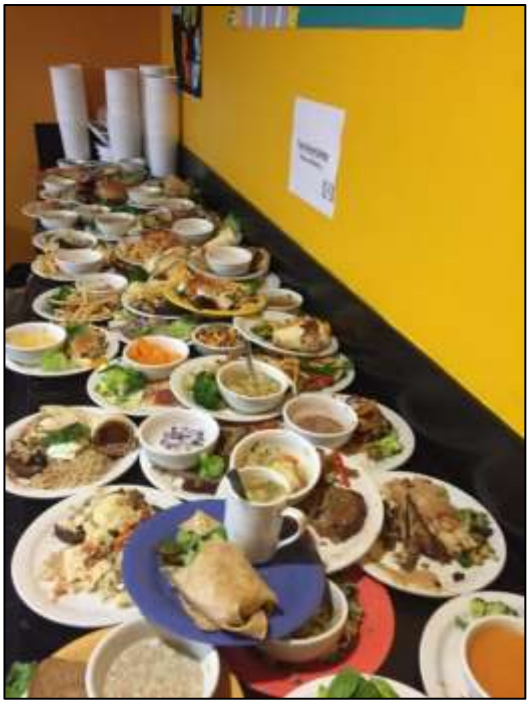

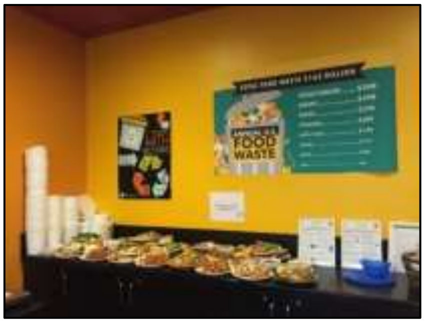 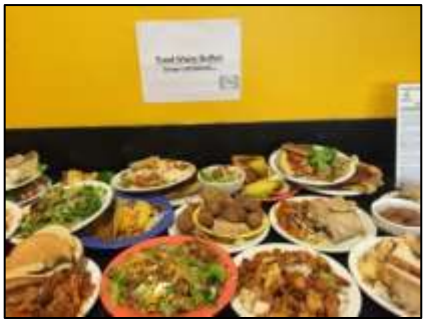

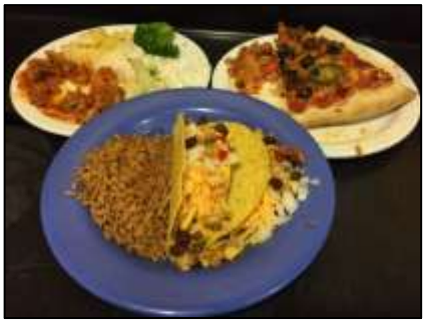 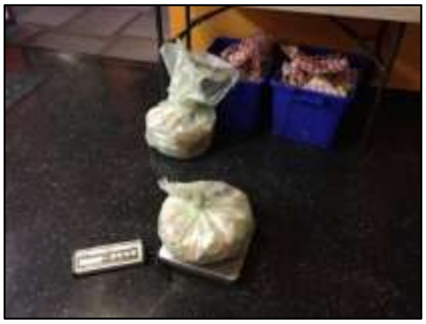

Sources:

UC Davis Love Food, Don't Waste Program: http://dining.ucdavis.edu/sus-recycling.html

UK Love Food Hate Waste Program: http://www.lovefoodhatewaste.com/

Program in the press:

Campus Sustainability Office. (2016, January 29). Portland State Inside PSU | News. Green Campus Spotlight. Portland State University, Portland, OR. Retrieved from https://www.pdx.edu/insidepsu/news/green-campus-spotlight-tackling-food-waste-one-lunch-time

Kennedy, M. (2016, February 17). Program shows Portland State students how much food they waste. Sustainability Initiatives. American School and University, Overland Park, KS. Retrieved from http://asumag.com/sustainability-initiatives/program-shows-portland-state-students-how-much-food-they-waste

Appendix A.2. Program signage

Appendix A.2.1. Wall signage

Resources developed by:

Crying food posters – Modified from *Love Food Hate Waste* program (permission to use images from program; information updated for USA)

The Food Waste Story – Developed by PSU Dining

Total Food Waste $162 Billion – Developed by PSU Dining

We're weighing the waste – Developed by PSU Dining

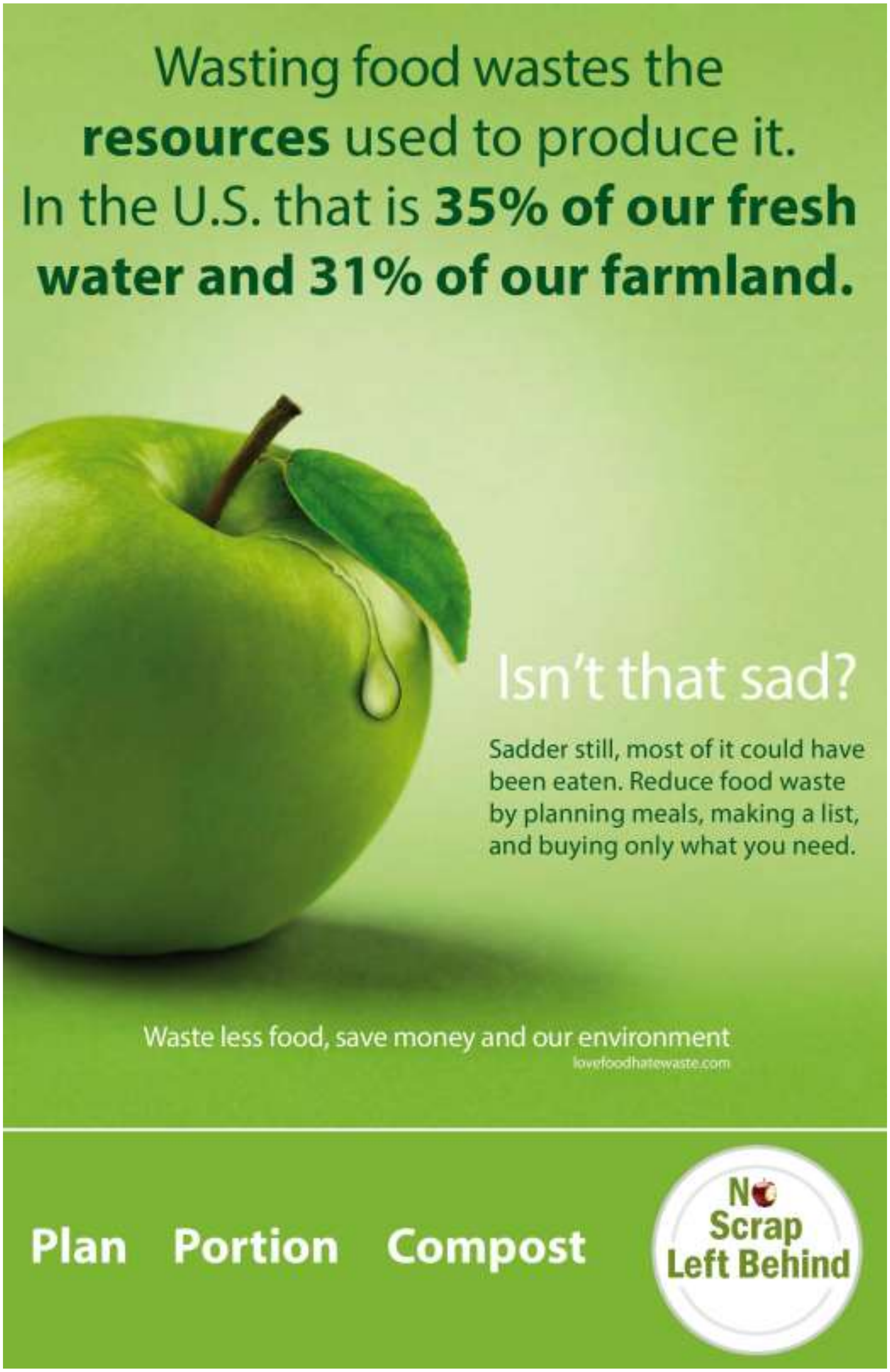
Wasting food wastes the **resources** used to produce it.
In the U.S. that is **35% of our fresh water and 31% of our farmland.**
Isn't that sad?
Sadder still, most of it could have been eaten. Reduce food waste by planning meals, making a list, and buying only what you need.
Waste less food, save money and our environment
lovefoodhatewaste.com
Plan Portion Compost
No Scrap Left Behind

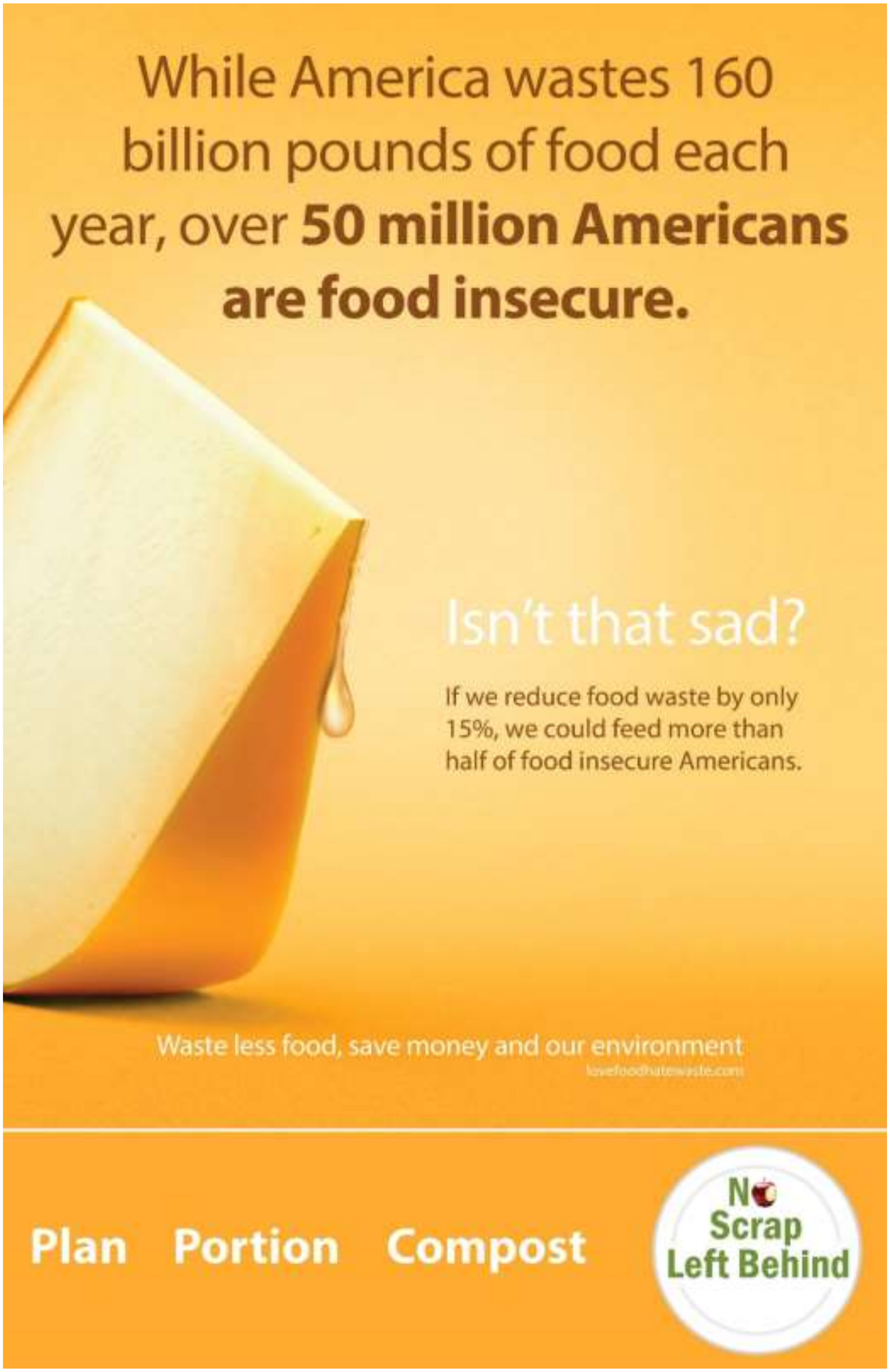
While America wastes 160 billion pounds of food each year, over **50 million Americans are food insecure.**
Isn't that sad?
If we reduce food waste by only 15%, we could feed more than half of food insecure Americans.
Waste less food, save money and our environment
Plan Portion Compost
No Scrap Left Behind

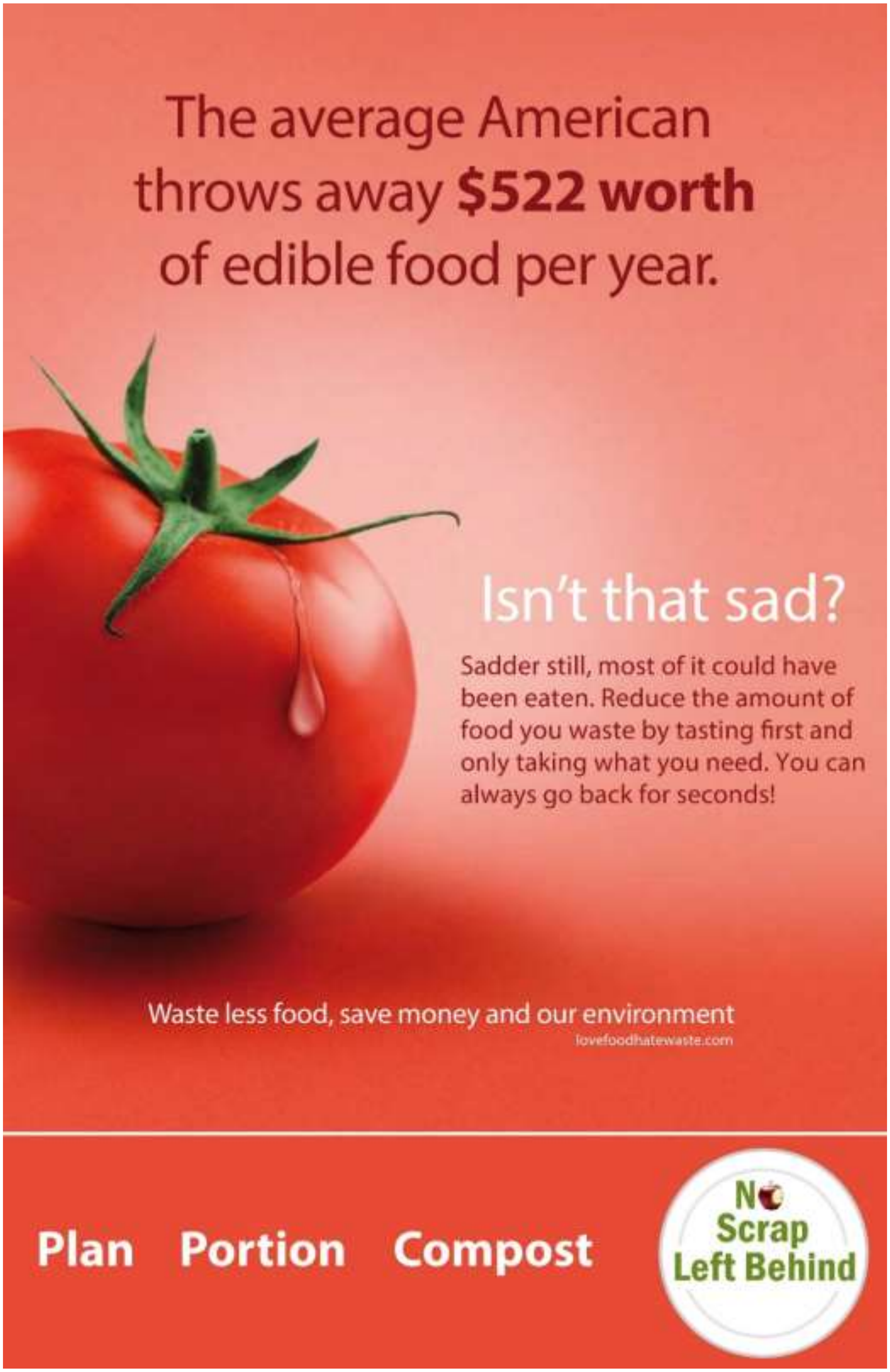
The average American throws away $522 worth of edible food per year.
Isn't that sad?
Sadder still, most of it could have been eaten. Reduce the amount of food you waste by tasting first and only taking what you need. You can always go back for seconds!
Waste less food, save money and our environment
lovefoodhatewaste.com
Plan Portion Compost
No Scrap Left Behind

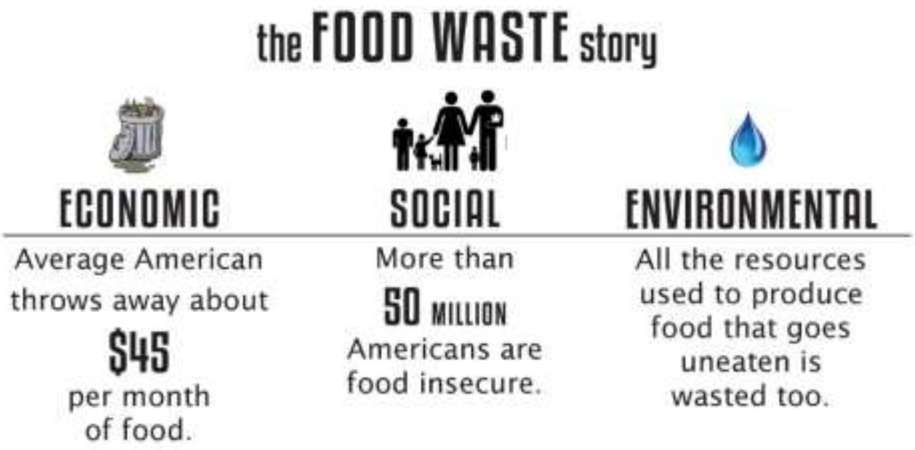

Bottom Line: Food Waste is a Colossal Issue.
But YOU can help address it!

What can YOU DO? It's EASY as 1,2 3!

1. PLAN	2. PORTION	3. COMPOST
shopping lists, use of leftovers, etc.	take and cook what you need.	recycle inevitable food scraps into rich soil.

No Scrap Left Behind!

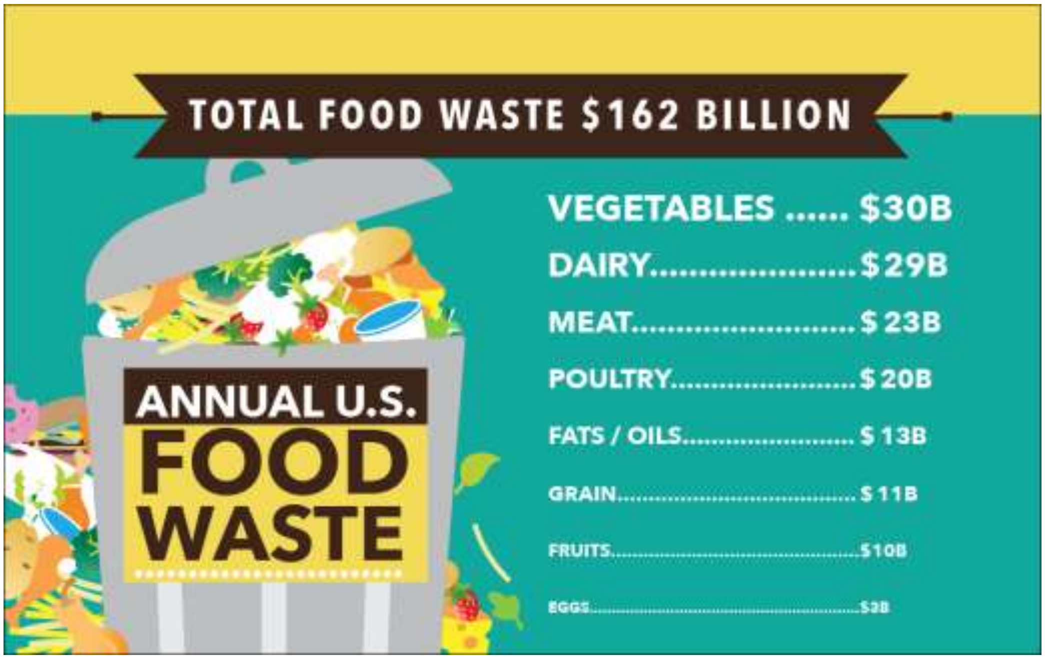
TOTAL FOOD WASTE $162 BILLION
ANNUAL U.S.
FOOD
WASTE
VEGETABLES $30B
DAIRY...................$29B
MEAT......................$ 23B
POULTRY...................$ 20B
FATS / OILS.................... $ 13B
GRAIN.................................$ 11B
FRUITS...$10B
EGGS...$3B

We're weighing the waste this week!

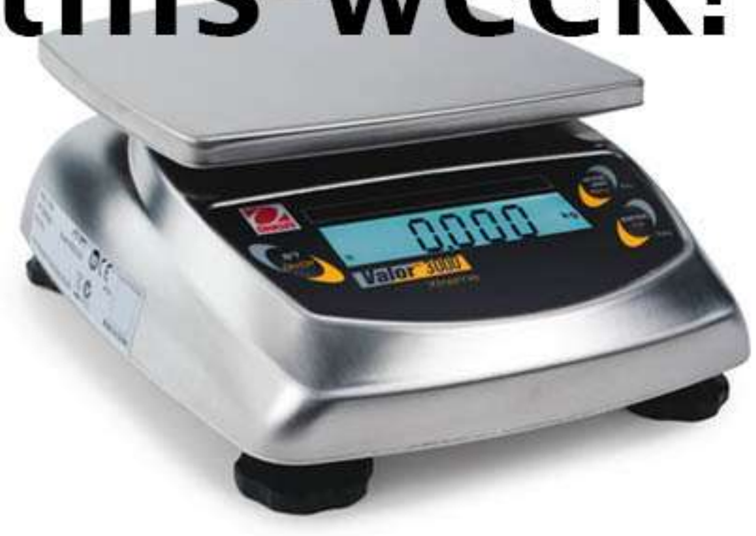

Appendix A.2.2. Napkin holder signage

Resources developed by:

Green napkin holders – Developed by Campus Sustainability Office

Food Waste Quizzes – Developed through coordinated efforts with all partners (Campus Sustainability Office (CSO), PSU Dining, Student Health and Counseling (SHAC), and Committee for Improving Student Food Security (CISFS))

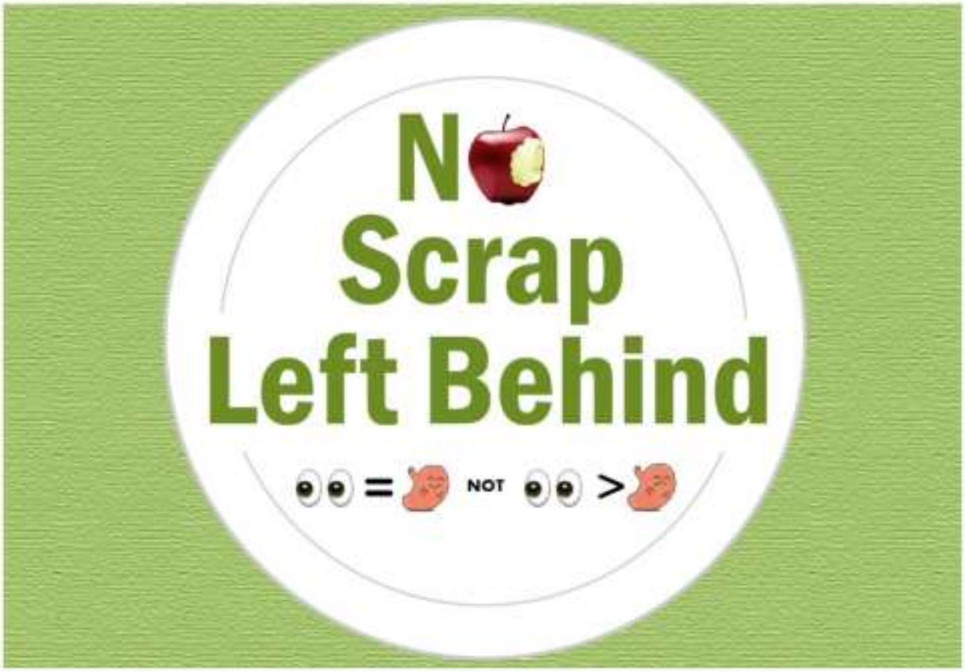
No
Scrap
Left Behind
NOT

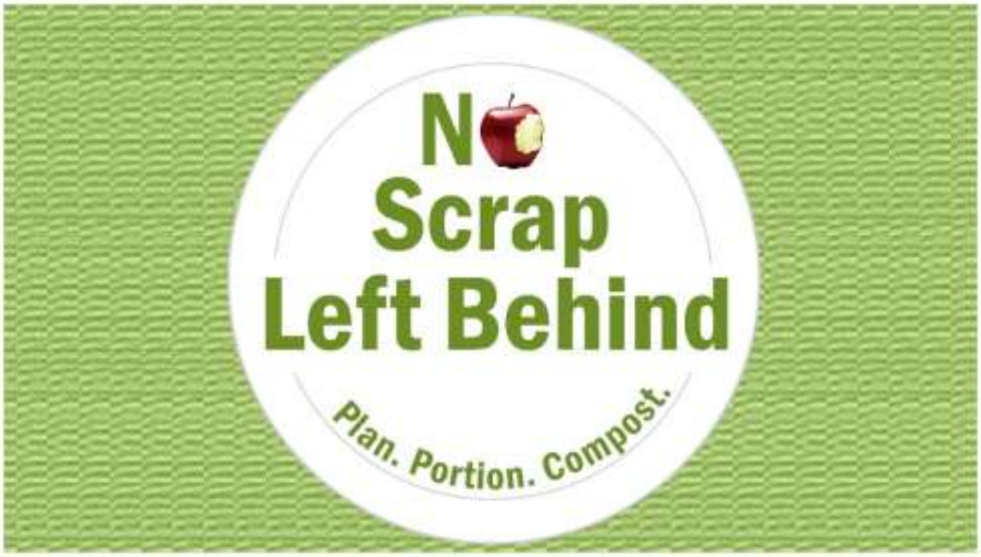
No
Scrap
Left Behind
Plan. Portion. Compost.

No Scrap Left Behind

Love Food. Hate Waste.

No Scrap Left Behind

Plan. Portion. Compost.

No Scrap Left Behind

Taste before you take.

No Scrap Left Behind

Think before you take.

No Scrap Left Behind

FOOD WASTE QUIZ

Grab a napkin, jot down some quick answers and get a prize as you leave today!

1. What types of food are the most wasted?
2. How many Americans are food insecure (do not know where their next meal is coming from)?
3. How many items can students receive (for free) by showing their PSU ID from the Student Food Pantry – now in the basement of SMSU?

No Scrap Left Behind

FOOD WASTE QUIZ

Grab a napkin, jot down some quick answers and get a prize as you leave today!

1. Per month, how much MONEY does the average American waste on food that goes uneaten?
2. What percentage of the food produced worldwide is estimated to go uneaten? TAKE A GUESS!
3. In just a few words, what is the meaning of "Food Security"?

No Scrap Left Behind

FOOD WASTE QUIZ

Grab a napkin, jot down some quick answers and get a prize as you leave today!

1. On average, how much food does ONE person waste in a month (pounds)? Just guess....
2. What material makes up the largest fraction of our total waste in the United States? (hint above)
3. Name one way YOU can cut down on food waste.

No Scrap Left Behind

FOOD WASTE QUIZ

Grab a napkin, jot down some quick answers and get a prize as you leave today!

1. What percentage of the food produced worldwide is estimated to go uneaten? It'll surprise you.
2. What is the name of the once a month opportunity for students to receive free fresh fruits & vegetables outside Shattuck Hall on the Park Blocks on the second Monday of every month at noon?

Quiz Key:

Yellow quiz

1. Fruits and veggies

2. About 50 million

3. Five per day

Green quiz

1. About $45

2. ~30%

3. Unsure if they will find their next meal

Blue quiz

1. ~20 lbs

2. Food

3. Many including: shop in bulk, eat/reuse leftovers, meal planning, portion correctly.

Purple quiz

1. ~30%

2. Harvest Share Free Market

Appendix A.3. Student handouts

Resources developed by:

Food Facts - Natural Resources Defense Council (NRDC) – Source: nrdc.org/policy

No Scrap Behind Tips! – Developed by Student Health and Counseling Center at Portland State University (SHAC)

FOOD FACTS

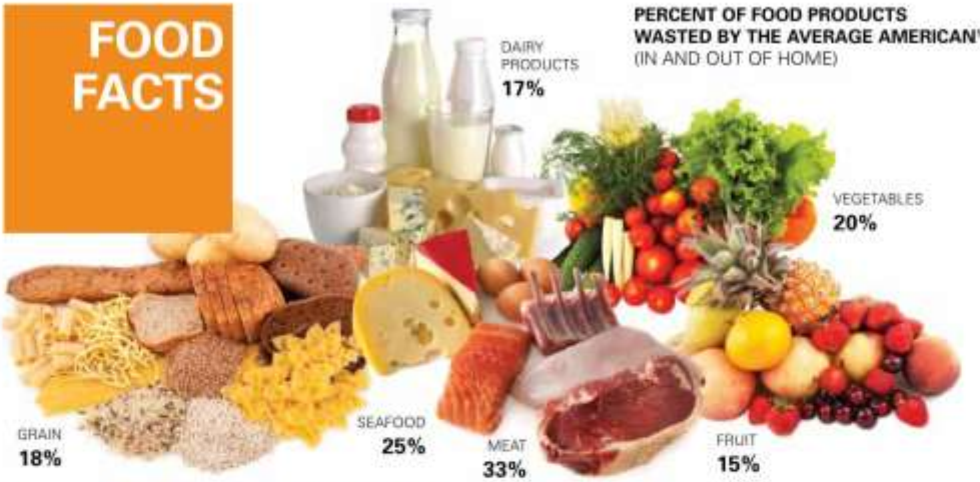

Your Scraps Add Up: Reducing food waste can save money and resources

Feeding the U.S. population requires an enormous amount of land and resources. Yet, 40 percent of food in the U.S. goes to waste. When the resources to grow that food are considered, this amounts to approximately 25 percent of all freshwater, 4 percent of the oil we consume, and more than $165 billion dollars all dedicated to producing food that never gets eaten. Reducing your own food waste is an easy way to trim down your bills and your environmental footprint.

HOW MUCH DO WE WASTE?

In the U.S., we waste around 40 percent[2] of all edible food. A large portion of that waste is caused by consumers. The average American throws away between $28-43[3] in the form of about 20 pounds[4] of food each month.If we wasted just 15 percent less food, it would be enough to feed 25 million Americans.[5]

Feeding the planet is already a struggle, and will only become more difficult with 9-10 billion people expected on the planet in 2050. This makes food conservation all the more important. The United Nations has predicted that we'll need up to 70 percent more food to feed that projected population.[6] Developing habits to save food now could dramatically reduce the need for increased food production in the future.

WHAT DOES WASTING FOOD COST US?

The cost of wasted food is staggering. In addition to the wasting of water, energy, chemicals, and global warming pollution that goes into producing, packaging, and transporting discarded food, nearly all of the food waste ends up in landfills where it decomposes and releases methane, a heat-trapping greenhouse gas that is 21 times more potent than carbon dioxide. Consider these cost estimates of all the food that never gets eaten in the U.S., and imagine just how much we can save by wasting less food:

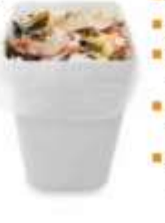

- 25 percent of all freshwater used in U.S.[7]
- 4 percent of total U.S. oil consumption[7]
- $165 billion per year[8] (more than $40 billion from households)[9]
- $750 million per year just to dispose of the food[10]
- 33 million tons of landfill waste (leading to greenhouse gas emissions)[11]

NRDC
THE EARTH'S BEST DEFENSE

For more information, please contact:

Dana Gunders
dgunders@nrdc.org
(415) 875-6100
switchboard.nrdc.org/blogs/dgunders/
Twitter: dgunders

www.nrdc.org/policy
www.facebook.com/nrdc.org
www.twitter.com/nrdc

25 million people could be fed if we reduced food waste by 15 percent

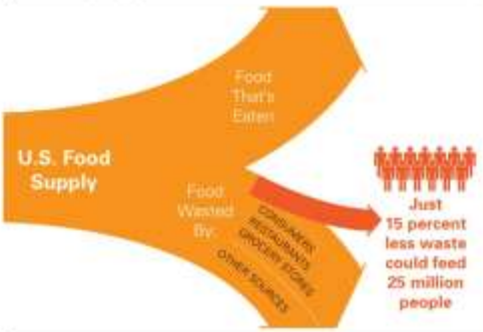

WHERE DOES FOOD WASTE COME FROM?

Food waste is a complex problem with losses occurring throughout the supply chain from "farm to fork." Crops are sometimes left unharvested because their appearance does not meet strict quality standards imposed by supermarkets. Food can be mishandled or stored improperly during transport. Large portions, large menus, and poor training for food handlers contribute to food waste in restaurants.

In households, fresh products make up most of the wasted food. The U.S. Department of Agriculture reports that a typical American throws out 40 percent of fresh fish, 23 percent of eggs, and 20 percent of milk. Citrus fruits and cherries top the list for fruits, and sweet potatoes, onions, and greens are commonly wasted vegetables.[12]

Much of household waste is due to overpurchasing, food spoilage, and plate waste. About 2/3 of household waste is due to food spoilage from not being used in time, whereas the other 1/3 is caused by people cooking or serving too much.[13] Single households produce proportionately more waste per person than multiple occupancy situations with more than one adult. Children, however, can add to the waste tally too. In fact, in a study of British households, those with children produced 41 percent more food waste than similarly sized households without children.[14]

The good news is we can reverse this costly food waste trend. Follow these tips and you'll finish your plate feeling satisfied in a whole new way.

EASY STEPS TO REDUCING YOUR FOOD WASTE

Follow these tips to keep your food bill and "food-print" down at the same time:

- **Shop Wisely**—Plan meals, use shopping lists, buy from bulk bins, and avoid impulse buys. Don't succumb to marketing tricks that lead you to buy more food than you need, particularly for perishable items. Though these may be less expensive per ounce, they can be more expensive overall if much of that food is discarded.
- **Buy Funny Fruit**—Many fruits and vegetables are thrown out because their size, shape, or color are not "right". Buying these perfectly good funny fruit, at the farmer's market or elsewhere, utilizes food that might otherwise go to waste.
- **Learn When Food Goes Bad**—"Sell-by" and "use-by" dates are not federally regulated and do not indicate safety, except on certain baby foods. Rather, they are manufacturer suggestions for peak quality. Most foods can be safely consumed well after their use-by dates.[15]
- **Mine Your Fridge**—Websites such as www.lovefoodhatewaste.com can help you get creative with recipes to use up anything that might go bad soon.
- **Use Your Freezer**—Frozen foods remain safe indefinitely. Freeze fresh produce and leftovers if you won't have the chance to eat them before they go bad.
- **Request Smaller Portions**—Restaurants will often provide half-portions upon request at reduced prices.
- **Eat Leftovers**—Ask your restaurant to pack up your extras so you can eat them later. Freeze them if you don't want to eat immediately. Only about half of Americans take leftovers home from restaurants.
- **Compost**—Composting food scraps can reduce their climate impact while also recycling their nutrients. Food makes up almost 13 percent of the U.S. waste stream, but a much higher percent of landfill-caused methane.[16]
- **Donate**—Non-perishable and unspoiled perishable food can be donated to local food banks, soup kitchens, pantries, and shelters. Local and national programs frequently offer free pick-up and provide reusable containers to donors.

Buzby, et al. The Value of Retail- and Consumer-Level Fruit and Vegetable Losses in the United States. Journal of Consumer Affairs, Fall 2011. 492-515.
Hall KD, Guo J, Dore M, Chow CC (2009) The Progressive Increase of Food Waste in America and Its Environmental Impact. National Institute of Diabetes and Digestive and Kidney Diseases. PLoS ONE 4(11): e7940.
Jonathan Bloom, American Wasteland (Cambridge: Da Capo Press, 2010).
Food and Agriculture Organization of the United Nations. *Global Food Losses and Food Waste*. http://www.fao.org/ag/ags/ags-division/publications/publication/en/?dyna_fef%5Buid%5D=74045.
This calculation assumes 2,500 kcal/capita/day and an annual total of 150 trillion calories in losses, as reported in K.D. Hall, J. Guo, M. Dore, C.C. Chow, National Institute of Diabetes and Digestive and Kidney Diseases. "The Progressive Increase of Food Waste in America and Its Environmental Impact," PLoS ONE 4(11):e7940, 2009.
Food and Agriculture Organization (FAO) http://www.fao.org/fileadmin/templates/wsfs/docs/expert_paper/How_to_Feed_the_World_in_2050.pdf.
See Hall 2009 above.
J. Buzby, and J. Hyman. "Total and per capita value of food loss in the United States", Food Policy, 37(2012):561-570.
Jones, Timothy. Corner on Food Loss. Biocycle, July 2005. p25.
USDA and US EPA. Waste Not, Want Not: Feeding the Hungry and Reducing Solid Waste Through Food Recovery. EPA 530-R-99-040. See vi above.
http://www.epa.gov/osw/conserve/materials/organics/food/fd-basic.htm.
http://www.ers.usda.gov/Publications/TB1927/TB1927.pdf.
http://www.wrap.org.uk/downloads/Household_Food_and_Drink_Waste_in_the_UK_Nov_2011.da3b2381.8048.pdf p27.
Ibid.
http://www.fsis.usda.gov/factsheets/food_product_dating/index.asp.
See USDA/EPA above.

NO SCRAP BEHIND TIPS!

What Should I Eat?

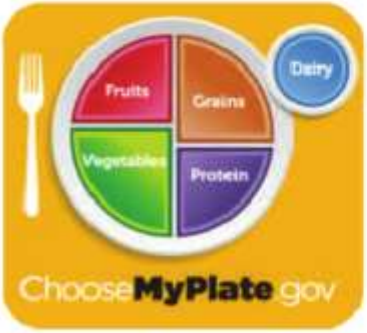

- Look for staple items in the **bulk section** like oatmeal, grains, and beans.
- **Frozen** veggies and fruits last longer and are nutritious.
- Check the **"day old"** bread section.
- **Re-use** yogurt , cottage cheese, & glass containers to store leftovers.
- Plan meals and make a grocery list – don't buy items you won't eat that week!

How much is one portion?

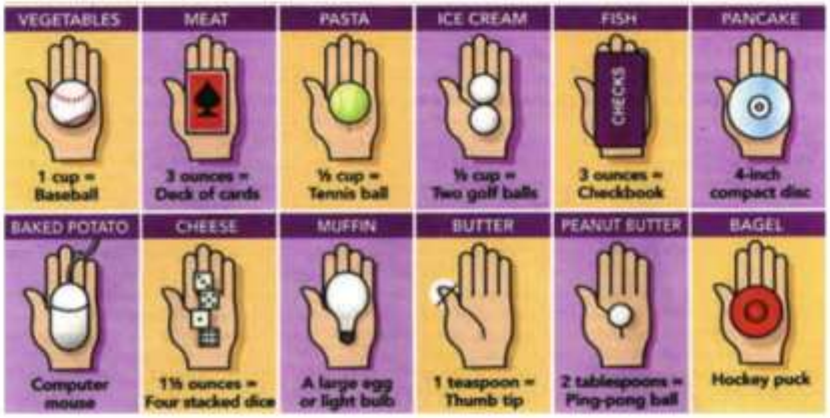

Learn more about portion sizes and how to reuse leftovers:

http://www.lovefoodhatewaste.com/recipes

Portland State UNIVERSITY

SHAC | Center for Student Health & Counseling
Medical | Dental | Counseling | Testing | Health Promotion

Peanut Butter and Jelly Granola Bars

Makes 12. Heat the oven to 350 °F. Butter or oil an 8" x 11" baking pan.

If you have a different size pan, that's fine—it'll just change how thick the bars are.

Pour the oats into a large bowl. You can use quick oats if they're all you have, but I prefer the bite and chew of rolled oats. For a different texture, you can also substitute a cup of oats with a cup of Rice Krispies, but the bars are great either way. Add the peanut butter, half the jelly, the water, and the salt to a small pan. Stir over low heat until it's smooth. Mix the peanut butter and jelly concoction into the oats until all the oats are coated and you have a sticky mass. Dump the mixture into the oiled pan and press it into an even layer. Spread the remaining jelly over the top. Pop the pan into the oven for 25 minutes, until it's toasty and brown around the edges. Mmm. Crunchy. Leave the bars in the pan until they cool completely, about an hour, then slice into 12 bars.

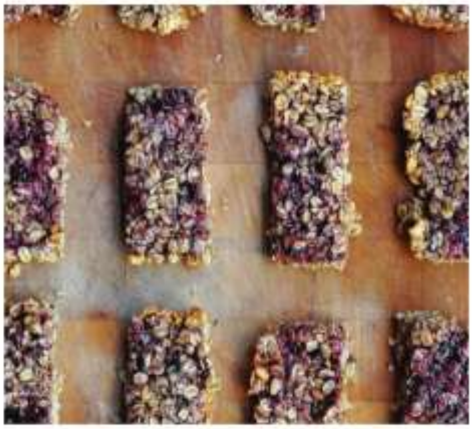

3 cups rolled oats (or 2 cups oats and 1 cup Rice Krispies)

½ cup peanut butter

½ cup jelly or jam

¼ cup hot water

¼ tsp salt butter or vegetable oil

Additions: nuts coconut dried fruit honey

EAT WELL ON $4/ DAY
GOOD AND CHEAP
To Download a Free PDF visit
ww.leannebrown.com

Check Out These Free Smartphone Apps!

Fridge Pal— helps you track what items are left in your fridge and expiration dates.

Food Planner—create your weekly shopping list, keep track of items, and build recipes.

Appendix A.4. Surveys

Resources developed by:

Short and long survey – part of dissertation work of Manar Alattar in partnership with other researchers, volunteers, and the CSO team.

Appendix A.4.1. Short survey (half page)

Food Diversion Survey

Please rate the following based on how strongly you agree or disagree with each statement:	Strongly agree	Agree	Neutral	Disagree	Strongly disagree
I think about the food waste I generate (Circle one)	1	2	3	4	5
My individual actions towards food waste do not make much of a difference	1	2	3	4	5
I put effort into reducing food waste	1	2	3	4	5
Food waste doesn't bother me	1	2	3	4	5
I eat leftovers	1	2	3	4	5
I check the refrigerator before shopping	1	2	3	4	5
I don't make lists and/or plan meals before shopping	1	2	3	4	5
I think about the portions of food that I take or cook	1	2	3	4	5
Composting contributes to the greater good	1	2	3	4	5
I compost my foodscraps	1	2	3	4	5
If I compost, I don't need to worry about source reduction (buying /preparing less food to avoid waste)	1	2	3	4	5
Composting stinks and is gross	1	2	3	4	5
Food breaks down in the landfill, so it doesn't bother me	1	2	3	4	5
I talk to other people about food waste	1	2	3	4	5
I understand food freshness labels (sell by, best by, use by, expiration date, etc.)	1	2	3	4	5

Food Diversion Survey

Please rate the following based on how strongly you agree or disagree with each statement:	Strongly agree	Agree	Neutral	Disagree	Strongly disagree
I think about the food waste I generate (Circle one)	1	2	3	4	5
My individual actions towards food waste do not make much of a difference	1	2	3	4	5
I put effort into reducing food waste	1	2	3	4	5
Food waste doesn't bother me	1	2	3	4	5
I eat leftovers	1	2	3	4	5
I check the refrigerator before shopping	1	2	3	4	5
I don't make lists and/or plan meals before shopping	1	2	3	4	5
I think about the portions of food that I take or cook	1	2	3	4	5
Composting contributes to the greater good	1	2	3	4	5
I compost my foodscraps	1	2	3	4	5
If I compost, I don't need to worry about source reduction (buying /preparing less food to avoid waste)	1	2	3	4	5
Composting stinks and is gross	1	2	3	4	5
Food breaks down in the landfill, so it doesn't bother me	1	2	3	4	5
I talk to other people about food waste	1	2	3	4	5
I understand food freshness labels (sell by, best by, use by, expiration date, etc.)	1	2	3	4	5

1. If I leave food on my plate the main reason is usually (choose one):
   a. It doesn't taste good
   b. I overestimated the portion size
   c. I'm being aware of caloric intake
   d. I don't have time to eat it
   e. I don't know
   f. Other ________________

2. Each year in the USA, over 160 billion pounds of food is wasted from production to consumption. What percentage of food do you think is lost at each step in the food-cycle? (Use table to answer)

**Production**	**Handling and Storage**	**Processing**	**Distribution and Market**	**Consumption**	Note: percentages should add up to 100%
%	%	%	%	%	

1. Everyone generates at least some waste. What *percentage* of food do you think is wasted by each of the following: (fill in below)

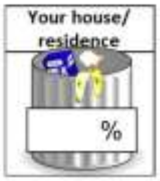

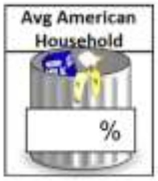

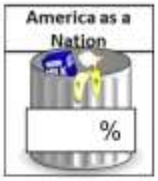

1. If I leave food on my plate the main reason is usually (choose one):
   a. It doesn't taste good
   b. I overestimated the portion size
   c. I'm being aware of caloric intake
   d. I don't have time to eat it
   e. I don't know
   f. Other ________________

2. Each year in the USA, over 160 billion pounds of food is wasted from production to consumption. What percentage of food do you think is lost at each step in the food-cycle? (Use table to answer)

**Production**	**Handling and Storage**	**Processing**	**Distribution and Market**	**Consumption**	Note: percentages should add up to 100%
%	%	%	%	%	

2. Everyone generates at least some waste. What *percentage* of food do you think is wasted by each of the following: (Fill in the below)

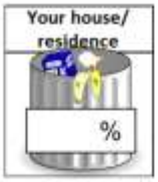

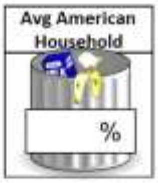

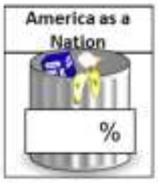

Appendix A.4.2. Full survey

Food Diversion Survey

1. Age: ____
2. Gender: ____
3. I am a (circle one): Student / Faculty / Employee
4. Do you live in a residence hall on campus? (Circle one) Yes / No
5. Are you enrolled in a freshman inquiry class that does waste audits? Yes / No
6. How many times have you taken this survey? ______
7. Do you participate in residence hall composting? Yes / No
8. How many times a week, on average, do you eat at PSU cafeterias, specifically:
    a. Victor's (Ondine)? _____
    b. Viking's (Smith)? ____
9. How many people (including yourself) live in your house, apartment or dorm-room? ____
10. Everyone generates at least some waste. What *percentage* of food do you think is wasted by each of the following: (Fill in the table)

Your house/ residence

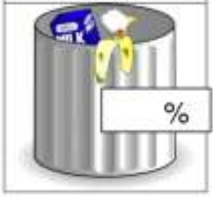

Avg American Household

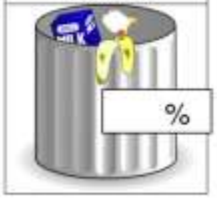

PSU Campus

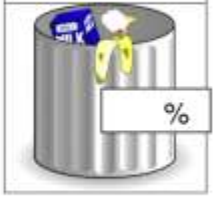

America as a Nation

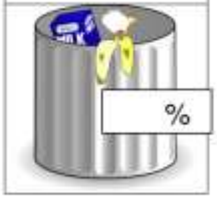

Please rate the following based on how strongly you agree or disagree with each statement:	Strongly agree	Agree	Neutral	Disagree	Strongly disagree
I think about the food waste I generate (Circle one)	1	2	3	4	5
I enjoy the food offered at Victor's dining hall	1	2	3	4	5
My individual actions towards food waste do not make much of a difference	1	2	3	4	5
I put effort into reducing food waste	1	2	3	4	5
I am interested in taking action to prevent food waste	1	2	3	4	5
Food waste doesn't bother me	1	2	3	4	5
I eat leftovers	1	2	3	4	5
I don't think the food I throw away costs much money	1	2	3	4	5
When I go to a buffet restaurant I take more than I can eat to get my money's worth	1	2	3	4	5
I check the refrigerator before shopping	1	2	3	4	5
I don't make lists and/or plan meals before shopping	1	2	3	4	5
I prepare/cook some of my meals	1	2	3	4	5
I think about the portions of food that I take or cook	1	2	3	4	5
I would be interested in attending a workshop on portioning or cooking for one person	1	2	3	4	5

Please rate the following based on how strongly you agree or disagree with each statement:	Strongly agree	Agree	Neutral	Disagree	Strongly disagree
I know about the residence hall compost program	1	2	3	4	5
Composting contributes to the greater good	1	2	3	4	5
I compost my foodscraps	1	2	3	4	5
If I compost, I don't need to worry about source reduction (buying /preparing less food to avoid waste)	1	2	3	4	5
I dislike compost and composting	1	2	3	4	5
Composting stinks and is gross	1	2	3	4	5
Food breaks down in the landfill, so it doesn't bother me	1	2	3	4	5
I talk to other people about foodwaste	1	2	3	4	5
I understand food freshness labels (sell by, best by, use by, expiration date, etc.)	1	2	3	4	5
I know about reusable to-go container options at Victor's	1	2	3	4	5
I use reusable to-go containers at Victor's	1	2	3	4	5
I believe that many materials can be reused or recycled into something new	1	2	3	4	5
I believe proper waste disposal makes a positive environmental impact	1	2	3	4	5
I believe that waste reduction and management is a potential career path or academic pursuit	1	2	3	4	5
I would enroll in a PSU course with a sustainability theme	1	2	3	4	5
I would like to see more programs at PSU that help reduce food waste.	1	2	3	4	5

11. If I leave food on my plate the main reason is usually (choose one):
    a. It doesn't taste good
    b. I overestimated the portion size
    c. I'm being aware of caloric intake
    d. I don't have time to eat it
    e. I don't know
    f. Other ________________

12. Each year in the USA, over 160 billion pounds of food is wasted from production to consumption. What percentage of food do you think is lost at each step in the food-cycle? (Use table to answer)

Production	Handling and Storage	Processing	Distribution and Market	Consumption	Note: percentages should add up to 100%
%	%	%	%	%	

13. What is your feedback on the cafeteria intervention program? Your feedback might say how the program affected you, what you learned or took away from it, or even how to improve it.

Appendix B. Food waste photo gallery program

***Food Left Behind* Art Gallery Project**

Background:
The *Food Left Behind* art gallery project was generated from ideas from various programs and student discussions about food waste. Specifically, in Portland State University's *No Scrap Left Behind* food waste diversion campaign it became obvious that the food waste buffet had an influence on students. On behalf of the Campus Sustainability Office along with other instructors and staff on campus, we connected with various food waste specialists (including Jordan Figueiredo, Dana Gunders, and Tristram Stuart) and it seems the idea of a food waste art show is new and could be developed upon beyond this program.

Introduction:
The food waste art show was developed from the available photo resources as well as student work from WALL-E classes to highlight the issue of food waste. The photos were displayed as it fits in the food cycle from production to consumption and waste.

Objectives:

4. To have students **reflect** on the process of wasting food and how it fits into the greater process
5. To **engage** the campus in food and food waste
6. To eventually produce a large, shareable **food waste art gallery** for universities.

Images of gallery May 31, 2016 Gallery

Photo credits: Lucas Powers, Jensine Tirado, and students. Images also provided by Ugly Fruits & Veg campaign.

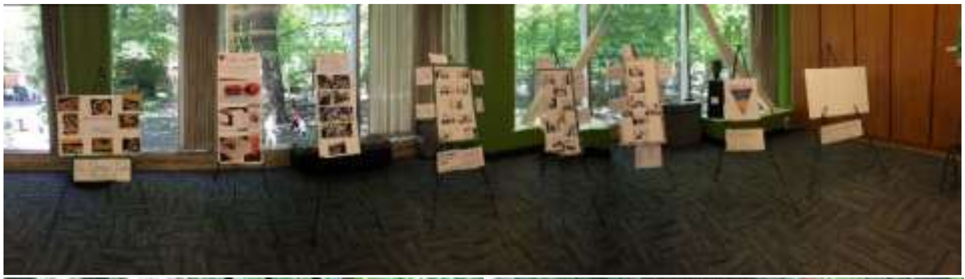

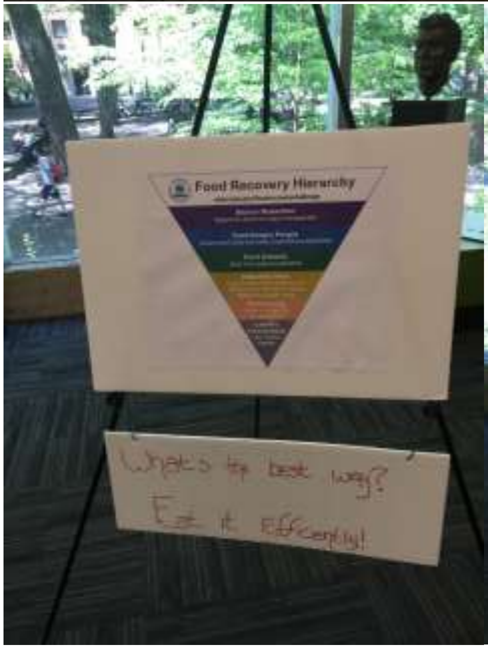
Food Recovery Hierarchy
What's the best way?
Eat it Efficiently!

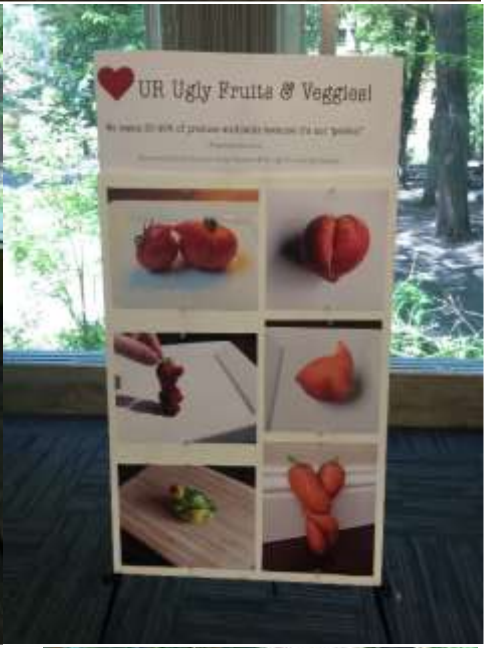
UR Ugly Fruits & Veggies!

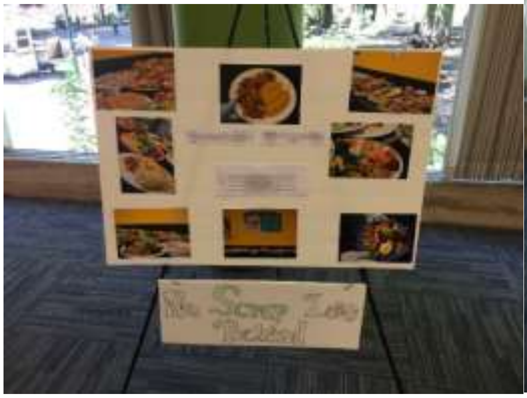

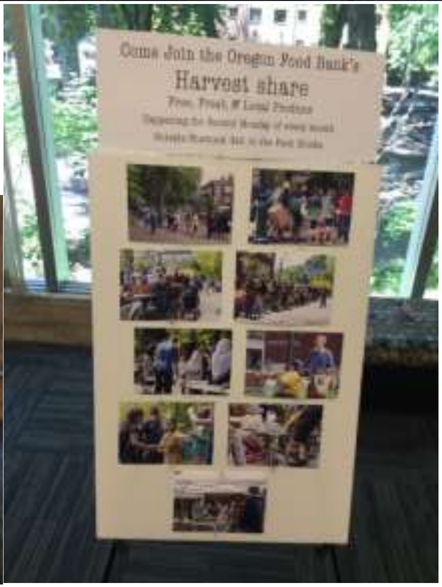
Come Join the Oregon Food Bank's
Harvest share
Free, Fresh, & Local Produce

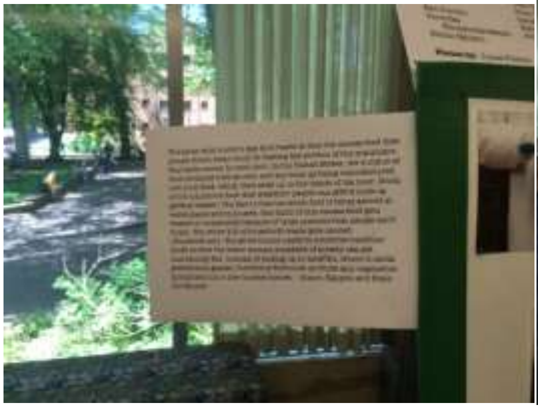

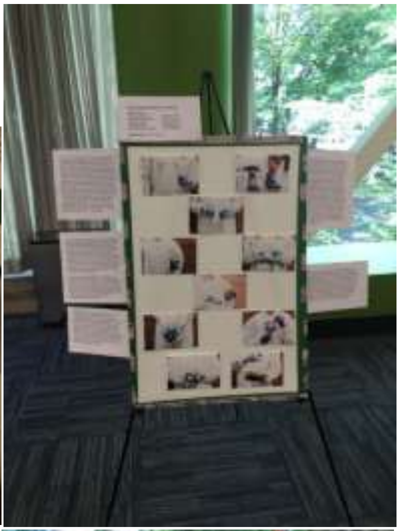

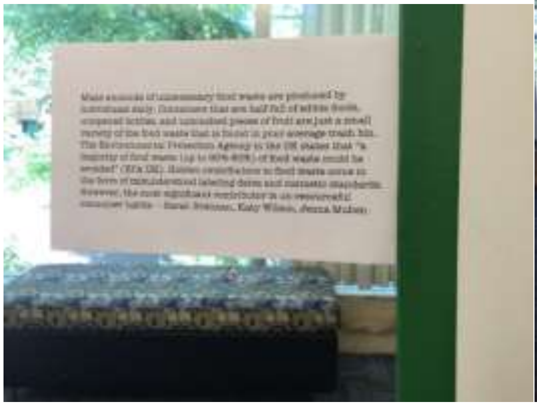

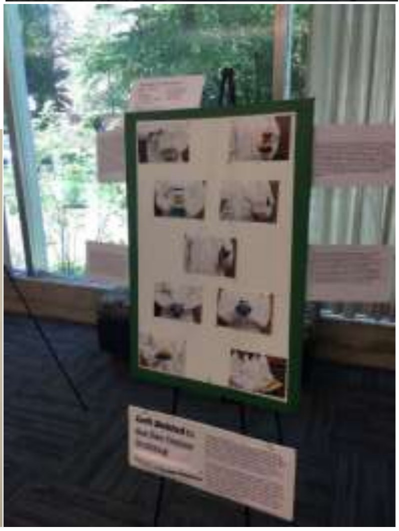

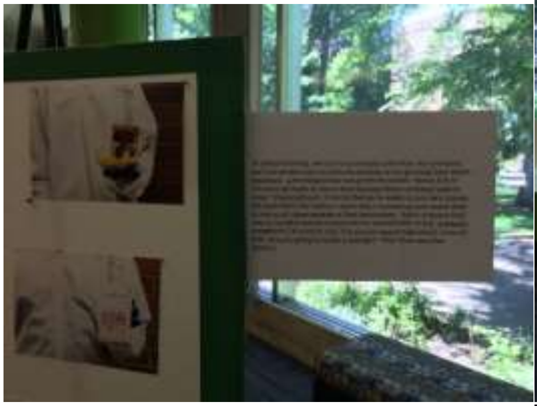
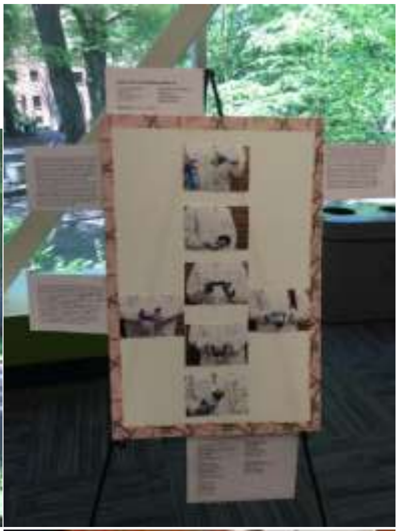
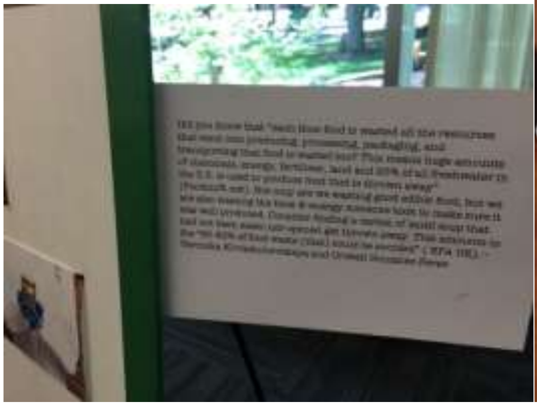
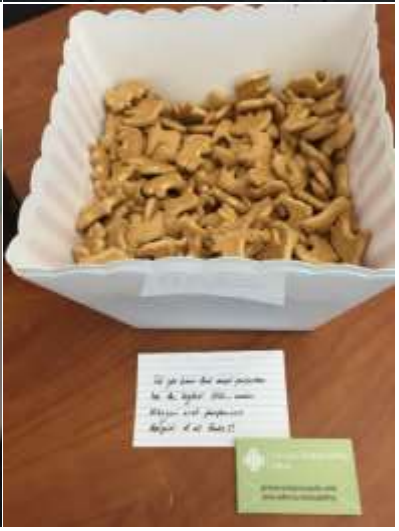

Appendix C. Food waste mindful cook-off activity

Food Waste Mindful Cook-off Activity

Name: *Cooking to Save the Planet!* OR *Cook Off the Scraps!*
(Other names may be appropriate as well depending on the venue)

Educational goal: To promote efficient kitchen and cooking habits that decrease food waste. The focus is on portioning, eating foods before they lose their shelf life, and utilization of left overs.

Overview of activity: Participants will cook a dish based on the following process and share it with the group. Dishes will be judged, by the audience, based on taste and display. Participants should be aware of the food waste associated with each dish. You can focus more or less on food preparation, food storage or food waste, depending on the event.

Guidelines:
1. Think of the top three kitchen ingredients that you often have trouble putting to use before they pass their optimal shelf life, be specific.
2. Use lovefoodhatewaste.com, www.bigoven.com/recipes/leftover or other resources to find a recipe that utilizes most of those items.
3. Cook and bring it to the event to be judged by the participants (based on taste and presentation).
4. You'll be asked to present your dish to the group.
5. Prizes for the top voted entree and dessert!

Note: cooking will be done at home and brought in potluck style.

Promotion: This activity is associated with a flier as well. See below for generic.

Campus Sustainability Office
Portland State University, Portland OR
Developed March 14, 2016

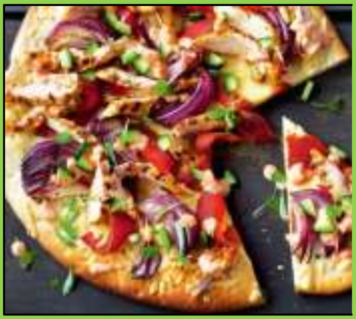

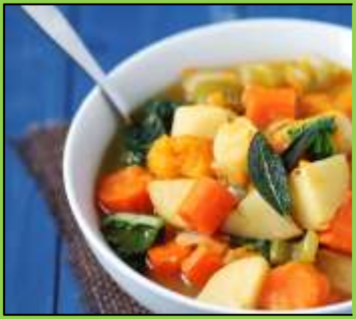

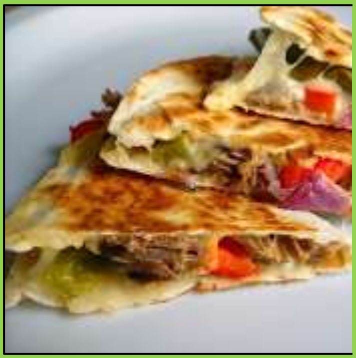

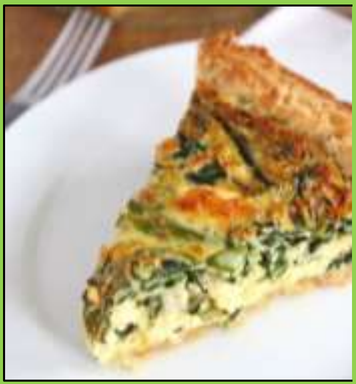

Image sources: www.taste.com.au/gallery/12+perfect+pizza+toppings,454; www.gimmesomeoven.com/slow-cooker-root-vegetable-stew/;www.budgetbytes.com/2010/02/roast-beef-quesadillas/;www.twopeasandtheirpod.com/asparagus-spinach-feta-quiche/

DATE

COOK OFF THE SCRAPS!!

Time – Location

SAVING FOOD SCRAPS ONE DISH AT A TIME

How can I compete?

1. Think of the top three ingredients that you often have trouble using before their optimal shelf life. Be specific.
2. Use lovefoodhatewaste.com, bigoven.com/recipes/leftover or other resources to find a recipe that utilizes most of those items.
3. Cook 'em up and bring your dish to the potluck to be judged by your colleagues. Judging criteria:
   - Taste; yay or nay?
   - Presentation; hot or not?
4. You'll also be asked to present your dish to the group.
5. **Prizes** will be awarded to the top voted *entree* and *dessert*!

Can I just come to eat?!

I'll be pretty hungry, but too busy to cook; can I still come?

Are you kidding me?! OF COURSE! The more voters and eaters, the less food waste! YAY!

Save the planet one pizza, stew, quesadilla, casserole, or quiche dish at a time!

Learn more about food storage and recipes that are amazing, easy and allow us to use up food before it loses its shelf life @ lovefoodhatewaste.com.

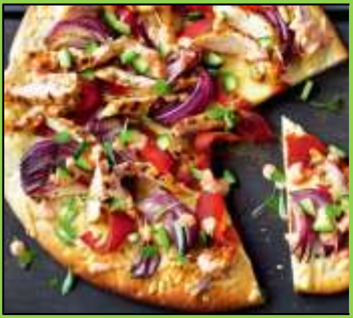

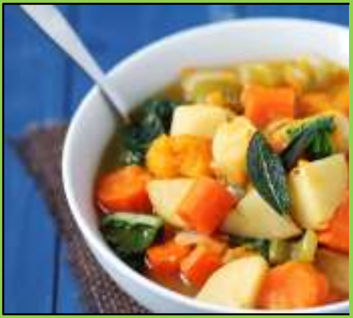

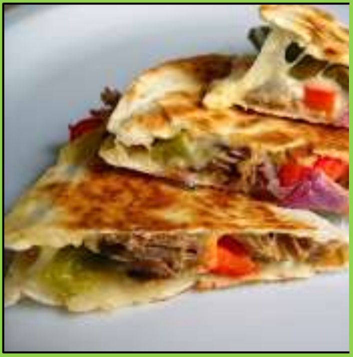

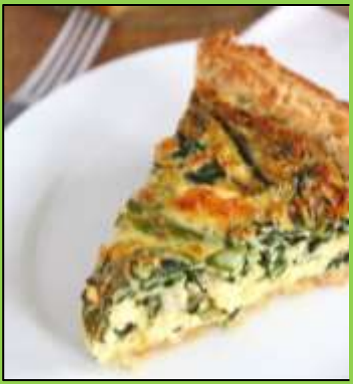

Image sources: www.taste.com.au/gallery/12+perfect+pizza+toppings,454; www.gimmesomeoven.com/slow-cooker-root-vegetable-stew/;www.budgetbytes.com/2010/02/roast-beef-quesadillas/;www.twopeasandtheirpod.com/asparagus-spinach-feta-quiche/

INSTRUCTIONS
HOW DOES THIS WORK??

1. Put a card near your dish with its name and a short description.
2. Taste and enjoy.
3. Read about the other dishes.
4. Write the number of the dish you like best (orange for sweet/green for salty) on a sticky, fold and insert it into the voting box.

Save the planet one pizza, stew, quesadilla, casserole, or quiche dish at a time!

Learn more about food storage and recipes that are amazing, easy and allow us to use up food before it loses its shelf life @ lovefoodhatewaste.com.
